# Symptomatic improvement after catheter ablation of supraventricular tachycardia measured by the arrhythmia-specific questionnaire U22

**DOI:** 10.3109/03009734.2010.517875

**Published:** 2011-02-11

**Authors:** Milos Kesek, Folke Rönn, Titti Tollefsen, Niklas Höglund, Ulf Näslund, Steen M. Jensen

**Affiliations:** ^1^Department of Cardiology, Heart Centre, University Hospital, UmeåSweden; ^2^Department of Thoracic Surgery, Heart Centre, University Hospital, UmeåSweden; ^3^Department of Medical Sciences, Umeå University, UmeåSweden

**Keywords:** Arrhythmia symptoms, catheter ablation, quality of life, supraventricular tachycardia, symptom-specific protocol

## Abstract

**Introduction:**

The main indication for ablation of supraventricular tachycardia is symptomatic relief. Generic measures of quality of life are not suitable for direct evaluation of arrhythmia-related symptoms, and a specific tool is needed. The questionnaire U22 quantifies symptoms associated with arrhythmic events. It uses discrete 0–10 scales for quantification of influence of arrhythmia on well-being, intensity of discomfort, type of dominant symptom, and a time aspect that summarizes duration and frequency of spells. We evaluated U22 in a well defined group of patients with paroxysmal supraventricular tachycardia, undergoing an intervention with a distinct end-point and a high success rate.

**Methods:**

Symptoms in patients with accessory pathway and atrioventricular nodal re-entrant tachycardia scheduled for ablation were measured with U22 and SF-36 on admission. The evaluation was repeated after 6 months.

**Results:**

Altogether 58 patients successfully ablated in 2006–2008 completed the four forms (U22 and SF-36 at base-line and follow-up, 210 ± 35 days after ablation). The score for well-being (0–10; 10 being best) increased from 5.9 ± 2.6 to 7.9 ± 1.9 (*P* < 0.0005). The score for arrhythmia as cause for impairment in well-being (0–10; 10 being highest) decreased from 7.5 ± 2.8 to 2.0 ± 3.1 (*P* < 0.0005). The time aspect score (0–10) decreased from 4.7 ± 1.5 to 1.4 ± 1.8 (*P* < 0.0005). The two SF-36 summary measures PCS and MCS increased from 46.9 ± 9.4 to 48.4 ± 10.7 and from 44.9 ± 12.5 to 49.1 ± 9.9 (*P* = 0.04 and 0.002).

**Conclusion:**

After successful ablation of accessory pathway and atrioventricular nodal re-entrant tachycardia, the U22 protocol detected a relevant increase in arrhythmia-related well-being. Modest improvement in general well-being was detected by the SF-36 protocol.

## Introduction

The main indication for ablation of supraventricular tachycardia (SVTA) is symptomatic relief (1). The success rate, judged immediately after the intervention, is known to be high (2–4). It has been shown, however, that even after a primarily successful ablation of accessory pathway many patients continue to suffer from arrhythmia symptoms (5). A more appropriate evaluation of procedural success therefore requires measurement of the symptoms at follow-up. The SF-36 (Medical Outcomes Study 36-Item Short-Form Health Survey) questionnaire and other general protocols measure quality of life and not the paroxysmal symptoms related to arrhythmia. Several arrhythmia-specific questionnaires have been described. The Symptom Checklist—Frequency and Severity Scale (6,7) has been used in ablation of SVTA, in revised versions in atrial fibrillation studies (8,9), and in a recent retrospective survey of late outcome after SVTA (10). The different implementations of the checklist are less well documented in the literature. Other questionnaires have been applied in atrial fibrillation (Symptom Specific Checklist with seven aspects of arrhythmia (11), Symptom Severity Questionnaire measuring five symptom parameters (12), a bedside-oriented questionnaire with classification into four groups (13)). None of these approaches fulfil the requirement of being well described and commonly accepted for use in different arrhythmia types.

U22 (Umea 22 Arrhythmia Questions) is a clinically oriented questionnaire, developed for evaluation of intermittent symptoms related to arrhythmia (see Appendix). The questionnaire quantifies multiple self-perceived symptom aspects associated with the arrhythmic events. In measurements on patients with SVTA scheduled for catheter ablation, U22 indicates a prominent discomfort during arrhythmia. Patients with SVTA that undergo catheter ablation are thus highly symptomatic, although their general well-being is only modestly decreased when measured by SF-36 (14). The SVTA ablation has a distinct end-point and a high success rate, making this patient group suitable for testing the questionnaire. In the present study, we investigate whether the arrhythmia-specific symptom questionnaire U22 is better suited than the generic questionnaire SF-36 for measurement of the clinical improvement after an ablation of SVTA.

## Material and methods

### Questionnaires

The arrhythmia-specific symptoms were measured with U22 (Swedish form) (14). The Appendix describes the questionnaire. SF-36, a well documented instrument (15), was used as a generic measure of quality of life. It quantifies the mental and physical well-being in eight subscales with a range of 0–100, together with a physical and a mental summary score.

### Patients

Patients with SVTA (accessory pathway (AP) and atrioventricular nodal re-entrant tachycardia (AVNRT)), admitted for scheduled routine catheter ablation at the Heart Centre, University Hospital, Umeå, Sweden during 2006–2008, were invited to answer the base-line U22 and SF-36 forms. Next day the diagnosis was established invasively and treated by catheter ablation. The evaluation with U22 and SF-36 was repeated 6 months after the ablation. The answers were prospectively entered into a database and retrieved for the subsequent analysis.

### Analysis

First-time interventions for the target diagnoses were selected. The catheterization reports from all ablations were reviewed by an experienced operator, blinded with respect to the U22 results (S.M.J.), and each procedure was evaluated for primary outcome. Ablation success in AP was defined as absence of delta wave and retrograde conduction block in the accessory pathway and in AVNRT as non-inducibility of tachycardia.

The data were analysed in SPSS, release 13 (SPSS Inc., Chicago, Illinois). The U22 and SF-36 scores were compared pairwise at base-line versus at follow-up in individual patients. Data are presented as mean ± SD. Differences between continuous variables were examined by paired and unpaired *t* test. Correlations (Spearman's rho) between individual patients' scores at base-line and follow-up were used for studying common properties of the responses in patients with varying symptomatology and perception of discomfort. Two-tailed Fisher exact test was used for testing differences in 2 × 2 table proportions. A *P* value < 0.05 was considered significant. The study was approved by the Ethics Committee at the Umeå University Faculty of Medicine.

## Results

### Effect of ablation on U22 and SF-36

**Figure 1.**
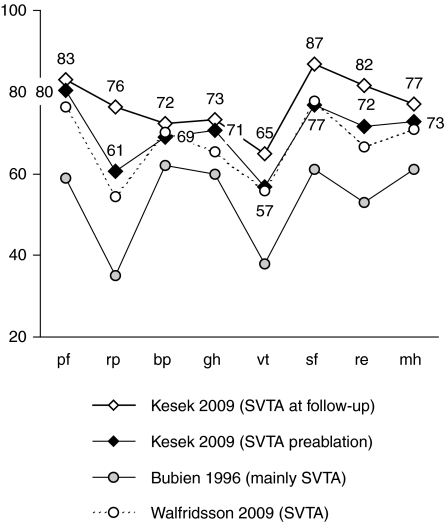
SF-36 subscales. The figure shows the SF-36 profiles at base-line and follow-up, labelled with mean values of the eight subscales in the study group. For comparison, the profiles of an older and a recently published group of patients with SVTA (6,18) are shown (the mean values were derived from the published graph and the number of patients). At base-line, the present study group is very close to the recent material by Walfridsson et al. (18). The profile is similar, but at a higher absolute subscale level than in the older material of patients by Bubien et al. (6). This may reflect a development towards ablation in patients with fewer symptoms. (pf = physical functioning; rp = role-physical; bp = bodily pain; gh = general health; vt = vitality; sf = social functioning; re = role-emotional; mh = mental health; SVTA = supraventricular tachycardia.)

Between April 2006 and May 2008, 141 patients underwent 156 ablations for SVTA (1.1 procedure/patient). From 63 patients undergoing first-time ablation for AP or AVNRT, all four forms were available (U22 and SF-36 at base-line and at the follow-up, 210 ± 35 days after ablation). Table I summarizes the background data. In 58 of these subjects the ablation was primarily successful, as judged by the blinded analysis. The results are summarized in Table II (see Appendix for definition of the U22 scores). According to the verbal definitions underlying the numerical score values, the arrhythmias were highly symptomatic at base-line, and a marked decrease in the symptoms was seen after a successful ablation. The U22 score for relevance of answers was high both at base-line and follow-up. The U22 score for general well-being (question 1) increased from 5.9 ± 2.6 at base-line to 7.9 ± 1.9 at follow-up (Table II). The score for patients' self-perceived improvement in well-being measured at follow-up (question 2) was 8.2 ± 2.1. The two SF-36 measures physical component summary (PCS) and mental component summary (MCS) increased after ablation from 46.9 ± 9.4 to 48.4 ± 10.7 and from 44.9 ± 12.5 to 49.1 ± 9.9 (*P* = 0.04 and 0.002). For the profile of the eight SF-36 subscales, see Figure 1.

**Table I.** Demographic data.

	Patients with all 4 forms (*n* = 63)		Patients with 1–3 missing forms (*n* = 78)	
	AP	AVNRT	AP	AVNRT
*n*	26	37	35	43
Age mean (SD), years	43.9 (17.9)	57.1 (14.0)	42.3 (18.4)	53.3 (17.4)
Men, *n* (%)	17 (65)	14 (38)	27 (77)	18 (42)

All four requested forms (U22 and SF-36 at base-line and follow-up) were available from 63 of the 141 patients undergoing first-time ablations for AP or AVNRT. Five of the 63 ablations with complete data were not primarily successful, and these patients were subsequently excluded. AP = accessory pathway; AVNRT = atrioventricular nodal re-entrant tachycardia.

**Table II.** U22 measures at base-line and follow-up.

	Question	Base-line	Follow-up	*P* (*t* test)
Well-being	q01	5.9 ± 2.6	7.9 ± 1.9	<0.0005
Arrhythmia events affecting well-being	q11	7.5 ± 2.8	2.0 ± 3.1	<0.0005
Discomfort during arrhythmia spell	q12	8.1 ± 2.2	2.3 ± 3.2	<0.0005
Symptom width^a^		2.5 ± 1.7	0.9 ± 1.4	<0.0005
Intensity of dominant symptom		9.9 ± 0.3	3.9 ± 4.6	<0.0005
Incidence of symptoms	q08	2.5 ± 1.1	0.9 ± 1.3	<0.0005
Time aspect^b^	q08, q10	4.7 ± 1.5	1.4 ± 1.8	<0.0005
Relevance score^a^	q19	9.6 ± 1.5	9.5 ± 1.7	n.s.

Data from 58 first-time ablations, classified as primarily successful. ^a^See Appendix for definition. ^b^See Appendix and Table III for definition. q01, q08, q10, q11, q12, q19 = U22 questions 1, 8, 10, 11, 12, 19.

**Table III.** Computation of the time aspect from the symptom duration and incidence scores.

			q08 How often					
			Never	On rare occasions	A few times a month	A few times a week	Daily	All the time
			0	1	2	3	4	5
q10 How long	Seconds	0	0	1	2	3	4	5
	Minutes, less than a quarter of an hour	1	0	2	3	4	5	6
	A quarter of an hour to one hour	2	–	3	4	5	6	–
	One to four hours	3	–	4	5	6	7	–
	Longer	4	–	5	6	7	8	9
	All the time	5	–	–	–	–	9	10

The time aspect is found by looking up the row with relevant symptom duration score (q08) and column with relevant symptom incidence score (q10). For most combinations the time aspect is a simple addition of the two scores (both with range 0–5). The combination of incidence ‘never’ and duration ‘minutes’ is, however, assigned time aspect value 0. The entries marked as ‘–’ are coded as missing values as they correspond to largely inconsequent combinations of the two scores: Incidence ‘never’ with durations ‘a quarter of an hour’ or more; incidences ‘few times a week’ or less with duration ‘All the time’; incidence ‘All the time’ with durations ‘a quarter of an hour’ to ‘four hours’. q08 = U22 question 8; q10 = U22 question 10.

### Correlation between U22 scores before ablation and at follow-up

The U22 score for well-being at follow-up correlated to the same score before the ablation (Spearman's rho 0.59; *P* < 0.0005). Similarly, a correlation was found in the time aspect score (rho = 0.32; *P* = 0.03). No significant correlations in the scores before the ablation and at follow-up were found for arrhythmia affecting the well-being (question 11), discomfort during a spell (question 12), intensity of dominant symptom, and width of symptoms (rho = 0.21, 0.12, 0.03, 0.06; *P* = n.s. for all). The score for well-being at follow-up was correlated to the pre-ablational symptoms of irregularity and anxiety during a spell (questions 15 and 21) (rho = -0.31 and -0.28; *P* = 0.03 and 0.04). An absence of correlation was noted between the patients' retrospective estimation of improvement in well-being after the ablation (question 2_follow-up_) and the improvement computed from the two forms as (question 1_follow-up_ – question 1_base-line_) (rho = 0.09; *P* = n.s.).

### Medication

At base-line, 71% of the patients did take some prescribed medication for the arrhythmia (57% in the AP group and 80% in the AVNRT group). At follow-up after a primarily successful ablation, 27% remained on medication (9% in the AP group and 38% in the AVNRT group).

In the two groups of patients on no anti-arrhythmic medication at follow-up (73%) and patients on continuing anti-arrhythmic medication (27%), the U22 score for well-being was 8.0 ± 1.8 versus 7.4 ± 2.2 (*P* = n.s. for the comparison). The scores for arrhythmia as a cause for impaired well-being were 1.5 ± 2.8 versus 3.3 ± 3.5 (*P* = 0.06), discomfort during attack 1.8 ± 2.9 versus 3.5 ± 3.7 (*P* = 0.09), dominant intensity 3.1 ± 4.5 versus 5.6 ± 4.8 (*P* = 0.09), width of symptoms 0.6 ± 1.1 versus 1.3 ± 1.8 (*P* = 0.1), and time aspect 2.2 ± 2.1 versus 0.9 ± 1.6 (*P* = 0.02). The SF-36 measures PCS and MCS were 50.6 ± 9.2 versus 42.8 ± 12.9 and 51.0 ± 7.8 versus 44.9 ± 13.6, respectively (*P* = 0.02 and 0.05).

## Discussion

We have evaluated U22 as a tool for measurement of arrhythmia-related symptoms in a well defined group of patients with paroxysmal supraventricular tachycardia who were undergoing an intervention with a distinct end-point and a high success rate. The scores quantifying the arrhythmia symptoms decreased notably. The improvement in general well-being was smaller. U22 thus seems to be more suitable for measuring the effect of ablation than a general questionnaire, like SF-36. In contrast to SF-36, the scales of U22 have a clinical arrhythmia-related meaning, making the questionnaire useful for clinical evaluation of changes in individual patients.

An important application for the U22 questionnaire may be in arrhythmias with more diffuse symptomatology and interventions with less well defined end-points, situations occurring in patients with atrial fibrillation (16,17).

### Correlation between U22 scores before ablation and at follow-up

The absence of correlation between base-line and follow-up in the scores describing the arrhythmia is reasonable, in view of a primarily successful ablation. Surprisingly, the retrospective estimation of improvement in well-being (question 2) was not related to the computed difference in well-being between base-line and follow-up. A patient's retrospective estimate of improvement after ablation therefore seems doubtful as a measure of ablation success.

### SF-36

The profile of the SF-36 subscales at base-line in our group resembles closely that of another recent Scandinavian group of patients with SVTA (18). Both groups have a similar profile to an earlier material (6), but on a higher subscale level (Figure 1).

A relation between general quality of life and the symptom-specific measures can be expected, since the arrhythmia-related symptoms are severe enough to motivate the patients to undergo ablation. This relation is rather weak in our material, where a marked decrease of symptom-specific measures is followed by a modest increase in the SF-36 measures. This is presumably due to the relatively high level of quality of life measured by SF-36 already at base-line, in spite of a marked arrhythmia-related symptomatology.

A surprisingly large proportion of our patients remained on medication at follow-up, in spite of a primarily successful ablation. It has been shown that, after a primarily successful ablation of accessory pathway, 39% of the patients continued to report arrhythmia symptoms and 8% remained on anti-arrhythmic treatment (5). Corresponding figures for the patients with accessory pathways in the present material are very similar, while an even larger proportion of patients with AVNRT remained on anti-arrhythmic medication. We lack information from ECG (Electrocardiographic)-monitoring that would confirm or exclude a relation of the measured symptoms to an arrhythmia. Therefore the reason behind the continuing need for anti-arrhythmic medication cannot be determined. Our AVNRT patients had a mean age of 57 years, 13 years more than the AP group. These middle-aged and elderly patients are often treated with beta-blockers or Ca-antagonists for hypertension. This is one possible explanation for the large proportion of patients that remain on medication after ablation of AVNRT.

### Limitations

The study describes the pattern of paroxysmal symptoms, measured by U22 in a group of patients before and after an ablation. However, we have no data on the validity and reproducibility of the questionnaire. For computation of specificity and sensitivity of the symptom measurement it would be necessary to relate the described symptoms to incidence of ECG-verified arrhythmia. However, no continuous rhythm monitoring was performed in the study.

Reference values for the U22 scores from some type of control group would be desirable. U22 aims at a type of specific symptom-focused measurement that makes it difficult to construct a proper control group with normal reference values. The U22 questions make sense for patients with arrhythmia symptoms or those that have been cured from an arrhythmia. To achieve meaning in normal controls the questions would have to be rephrased. In the present form it is, however, reasonable to view the U22 scores of the subgroup free from medication at follow-up as representing a reference level after a successful treatment, since 1) the indication is based on a combination of symptoms and invasive electrophysiological diagnosis, and 2) the treatment can be considered as successful in patients that have been primarily successfully ablated and have no anti-arrhythmic medication at follow-up. The values of the SF-36 parameters PCS and MCS in this group are very close to 50 (normal).

A large proportion of patients had to be excluded from the analysis, due to failure to return all the four forms. The number of forms requested from each patient was demanding. Two additional factors may have contributed to the high exclusion rate: the base-line forms were not collected by specifically assigned staff, but as a part of normal patient admission routines; and in case of missing follow-up forms, no reminder was sent out. The patients that were excluded due to missing forms were slightly younger, with a higher proportion of men (Table I).

The English version of U22 was translated from Swedish with the intention of corresponding closely with the original meaning. A translation nevertheless introduces a source of error into any comparisons. Comparison of treatments based on different translations of a questionnaire should therefore be accompanied by some evaluation, at least by a comparison of the initial levels in similar populations.

## Conclusion

The U22 questionnaire detected the expected symptomatic improvement in patients ablated with primary success for AP and AVNRT. A prominent increase could be seen in measures of arrhythmia-related well-being. In comparison, the improvement observed in the generic SF-36 questionnaire was relatively small.

## Appendix

### U22 questionnaire

The U22 instrument aims at quantifying prevalence and severity of arrhythmia-associated symptoms (14). It consists of two related questionnaires to be answered at base-line and follow-up after an intervention, respectively.

Questions about general well-being, the influence of arrhythmia on the well-being, and the intensity of discomfort and specific symptoms during a spell are answered by choosing one of 11 alternatives, aligned in a horizontal line of 10 cm length, with verbal descriptions of the end-points (anchors). The subject is instructed to select the answer by placing an X in the circle that best reflects how he feels. The answers are translated to a discrete numeric rating scale with range of 0–10 (NRS-10) (Numerical Rating Scale, a discrete analogy to the common VAS [Visual Analogue Scale]).

The scores give an estimate of well-being, the effect of arrhythmia events on the well-being, discomfort during a spell, and the maximal symptom intensity. A symptom width score is computed as the number of dominant symptoms (the symptoms rated as having equal, maximal intensity). The symptom width score is set to 0 if the subject disagrees to some degree with all symptom descriptions, i.e. all the scores from questions 11–22 are <5. The subject's understanding and relevancy of the answers is tested by question 19 about itching, a symptom irrelevant in the context of arrhythmias. A relevance score is computed as (10 – scale 19).

The incidence and duration of the events is estimated by questions 8 and 10 with 6 non-linear answer alternatives, quantified as 0–5. A time aspect score with a range of 0–10 is computed by adding the two answer scores (Table III).

#### Base-line form (English translation of the original Swedish forms used in the study)

The answer choices are stated within the square brackets.

**Table:**

1.	On the whole, how have you felt over the past month?	[Miserable–very well, 0–10]
	(2 – not applicable in base-line form)	
3.	Do you take any prescribed medications for your heart rhythm problems?	[No; Yes]
4.	How effective is the medication(s) in treating your heart rhythm problems?	[Very much worse–very much better, 0–10]
5.	How much are you bothered by side-effects of the medication?	[Not at all–very much, 0–10]
	(6, 7 – not applicable in base-line form)	
8.	How often do you experience problems with heart rhythm? (despite taking any medication)	[Never; Rarely; A few times a month; A few times a week; Daily; All the time]
9.	Do the spells start suddenly?	[No; Yes]
10.	How long does a spell usually last?	[Seconds; More than a minute but less than 15 min; 15 min to 1 hour; 1 to 4 hours; More than 4 hours; All the time]
11.	How much do the spells affect your well-being?	[Not at all–very much, 0–10]
12.	How bothered are you while you are experiencing a spell?	[Not at all–very much, 0–10]
13.	My heart beats extremely fast when I'm having a spell	[Not at all–very true, 0–10]
14.	My heart pounds very heavily when I'm having a spell	[Not at all–very true, 0–10]
15.	My heart beats very irregularly when I'm having a spell	[Not at all–very true, 0–10]
16.	I faint or feel extremely dizzy when I'm having a spell	[Not at all–very true, 0–10]
17.	I have terrible pain when I'm having a spell	[Not at all–very true, 0–10]
18.	I feel extremely tired when I'm having a spell	[Not at all–very true, 0–10]
19.	I feel like my skin itches all over when I'm having a spell	[Not at all–very true, 0–10]
20.	I feel extremely short of breath when I'm having a spell	[Not at all–very true, 0–10]
21.	I become very frightened when I'm having a spell	[Not at all–very true, 0–10]
22.	Someone close to me becomes very frightened when I'm having a spell	[Not at all–very true, 0–10]

##### Follow-up form

The follow-up questionnaire is equivalent to the base-line form, with an addition of three questions that explore the type of remaining symptoms (question 6) and the present general well-being and arrhythmia symptomatology in comparison to base-line (questions 2 and 7).

**Table:**

2.	Compared to the time before the treatment, do you now feel:	[Very much worse–very much better, 0–10]
6.	Have you experienced any problems with the heart rhythm after the treatment? (Please disregard the first 3 months after the treatment.)	[No; Yes, of the same type as before the treatment; Yes, of different type]
7.	How bothersome are the spells in comparison with those before the treatment?	[Very much more–very much less, 0–10]

Questions 7–22 in the follow-up form are only answered if the patient in question 6 indicates any remaining problems with the heart rhythm. If the patient has no such problems, no further answers are requested, and on analysis the scores for questions 8 and 10–22 will be set to 0, while question 7 will be set to 10.
